# Diversity of beetles (Coleoptera) in natural and planted saxaul forests (*Haloxylon ammodendron*) in the South Gobi Desert, Mongolia

**DOI:** 10.3897/zookeys.1000.56856

**Published:** 2020-12-03

**Authors:** Buyanjargal Batchuluun, Jens Wunderlich, Michael Schmitt

**Affiliations:** 1 Laboratory of Entomology, Institute of Biology, Mongolian Academy of Sciences, Ulaanbaatar, Mongolia Institute of Biology, Mongolian Academy of Sciences Ulaanbaatar Mongolia; 2 Michael Succow Foundation, Greifswald, Germany Michael Succow Foundation Greifswald Germany; 3 General and Systematic Zoology, Zoological Institute and Museum, University of Greifswald, Greifswald, Germany University of Greifswald Greifswald Germany

**Keywords:** Beetle community, black saxaul, species richness

## Abstract

We investigated species composition and diversity parameters of beetle communities in two planted saxaul (*Haloxylon ammodendron*, black saxaul) forests in Southern Mongolia. We also studied nearby natural areas for comparison. Beetles were mainly collected by pitfall traps. 1064 individuals of 38 species of 22 genera in 4 beetle families were identified from planted plots. In comparison, a total of 1395 beetles belonging to 40 species of 24 genera in seven families were collected and identified from the natural saxaul plots. The most diverse beetle families were darkling beetles (Tenebrionidae, 18 species) and snout beetles (Curculionidae, 15 species) in planted and natural saxaul plots. We recorded several species (*Apatophysis serricornis*, *Cephogenia chinensis*, and *Eumylada punctifera punctifera*) which are associated with the saxaul tree. A darkling beetle, *Anatolica potanini*, was the dominant species in both natural and planted plots of the Nariin Zag forest. There were significant differences in the species richness and abundance between the planted and natural plots of the Ukhaa Zag forest. It is possible that the age of the plantation drove the differences. The higher values of diversity indices and species richness in the planted plots can be explained by the presence of rare species, represented by only one or two individuals. The planted plots and corresponding natural plots within each forest were more similar to each other in species composition and abundance than between forests.

## Introduction

Beetles constitute the main component of insect communities in arid landscapes. They inhabit these landscapes in close interaction with plants, using them as food resources, for shelter, and as development sites. Beetles are regarded to be indicators of environmental changes (e.g., Jennie and Lisa 2006; Jake et al. 2014; Samir et al. 2018) and changes in their diversity might reflect environmental changes in natural habitats, corresponding to changes in host plant communities.

Insect biodiversity studies in arid regions of Mongolia have a goal of understanding the current state of the ecosystem, which suffers from the pressure of human activities, including livestock overgrazing and mining operations. Climate change is an additional stress factor, as it is occurring at a much higher rate in Mongolia than the global average, particularly in the Gobi Region (Damdin et al. 2014). Essential for conservation of those interacting organisms that define the Gobi’s natural appearance is the identification of insect species, especially beetles, which exhibit particular adaptation mechanisms to arid ecosystems, associated with saxaul (*Haloxylon ammodendron*, black saxaul). Saxaul is one of the most important and useful native plants in the arid region that extends from Iran and Central Asia eastward across the Gobi. Only in Southern Mongolia does saxaul grow as forests. A saxaul forest sustains a distinct environment of plants, animals, microclimate conditions, and soil in the Gobi. Moreover, it controls desertification in several ways by helping to fix shifting sands, increasing biological productivity of arid areas, and restoring degraded pasture and forest (Orlovsky and Birnbaum 2002). Therefore, there are several efforts to plant saxaul in the South Gobi province of Mongolia.

Restoration success of natural habitats can be evaluated using their beetle communities as indicators of overall habitat quality (Cárdenas et al. 2011; Fattorini and Salvati 2014; Januschke and Verdonschot 2016; Steiner et al. 2016).

The purpose of the present study is to investigate the diversity of the beetle community in natural and planted the saxaul forests and to identify whether or not planted forests support the natural beetle community.

## Method

### Study area

The study was carried out in the Umnugovi Aimag, the South Gobi province of Mongolia. The climate of the area is typically continental with cool spring and autumn, warm dry summer, and cold winter. The average maximum temperature in July is 31.1 °C; the average minimum temperature in December and January is −15.0 °C. The average annual total precipitation is 97 mm, and the average number of annual rainfall days is 32 days (averaged from the nearby “Khanbogd” weather station, using data from 1976–2014). According to the classification of phytogeographical regions of Mongolia, the study area belongs to the desert steppe of the East Gobi Region (Grubov 1954). The soil in the study sites is dominated by saline Gobi gray-brown soil.

### Study sites

**Figure 1.**
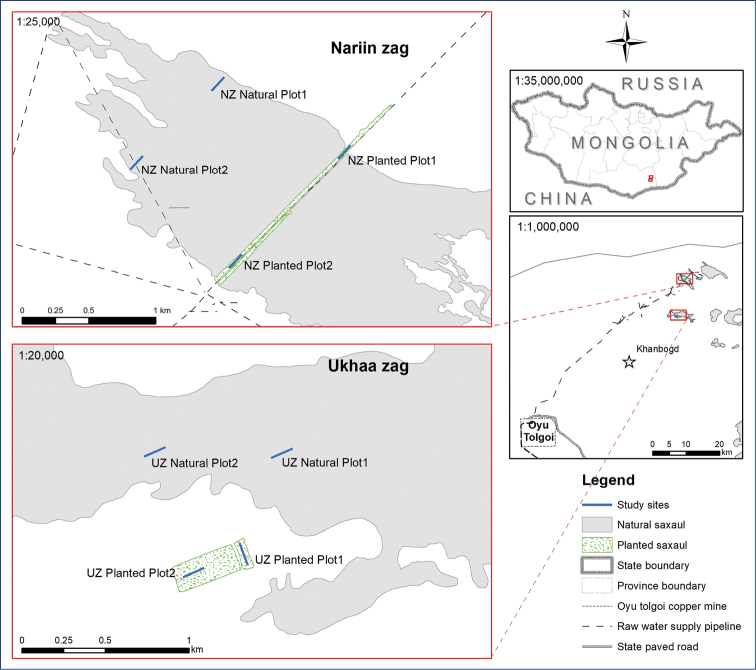
Study sites and plots.

In accordance with the environmental rehabilitation and offset programs of Oyu Tolgoi Limited Liability Company (LLC), the biggest copper mining company in the south Gobi province of Mongolia, saplings of *Haloxylon ammodendron* (black saxaul) were planted in two different saxaul forests:

1. Nariin Zag site (NZ). The “Nariin Zag” forest is located in the “Gunii Khooloi” depression of the Galba Gobi. Its area is about 564 ha. During the construction work on the “Gunii Khooloi” water pipeline of Oyu Tolgoi project in 2011, a 12.3 ha area with 8,112 saxaul individuals was destroyed. To reduce the impact, 10,677 individuals of two-year-old the saxauls were planted in 2013 in a 6.34 ha area. Its growth success was 60–88% in 2016 (Oyu Tolgoi LLC 2018). We chose two natural (not planted) saxaul plots as a control group and two planted plots for beetle community studies. The study plots were situated in the middle part of the forest where there are low hills with sandy loam soil. The main vegetation consists of Haloxylon ammodendron,Cleistogenes songorica, Salsola laricifolia, and Aristida heymannii. The average height of the saxauls is around 1 m.
2. Ukhaa Zag site (UZ). The “Ukhaa Zag” forest covers 812 ha and is also located in the Galba Gobi. In 2016, 5,508 two-to-three-year-old saplings were planted in 7.6 ha to enhance the area of the forest (Oyu Tolgoi LLC 2016). Our study site was in an open landscape with loose and thick sandy soil. The saxauls there are short (average 60–80 cm) and scattered. The secondary plants are Peganum nigellastrum and Nitraria sibirica. In the forest, we chose two natural (control) and two planted plots (Fig. 1).

### Sampling methods

We set 15 pitfall traps in each plot along a line. The distance between the traps was 10 m. The sampling interval was 7 days and traps were active 24 hours a day for each sampling interval. We used empty pitfall traps to avoid attracting animals by water. In addition to the pitfall traps, we beat and shook 10 saxaul trees every 14 days for plant dwelling beetles and subsequently searched the sand under the trees.

Sampling was carried out during the plant growing season from June to September in 2019. Beetles were preserved in 70% ethyl alcohol and were subsequently transported for further processing and species identification to the Laboratory of Entomology, Institute of Biology, Mongolian Academy of Sciences (MAS) (e.g. Baitenov 1974; Ter-Minasian 1979; Medvedev 1990).

### Statistical analysis

Observed species richness (Mao Tau), Pielou’s evenness index, and Shannon and Simpson diversity indices were used as diversity parameters. Paired T-tests were used to compare beetle diversity, species richness, and abundance differences between natural and planted plots. We calculated a Bray-Curtis index for cluster analysis. Diversity and similarity analyses were done by EstimateS 9.1.0 and paired T-tests calculated in Minitab Stat 15. Significance level was 0.05.

Beetles from the tree shaking samples were not analysed statistically, only searched for additional species. The distribution range analysis was done for captured species using an approach in Buyanjargal et al. (2016).

## Results

A total of 3,349 beetles belonging to 48 species of 30 genera in eight families were identified from natural and planted saxaul plots. Therein, 1,395 beetles belonging to 40 species were collected and identified from natural saxaul plots and 1,064 individuals of 38 species were identified from planted plots (Appendix 1).

According to the distribution range analysis, the majority of species (17 species, 37.0%) are distributed only in northern China and southern Mongolia, and eight species (17.4%) are endemic to Mongolia. Other species have a wider range from Kazakhstan to southern China (4.3%), southern Siberia, and Mongolia (8.7%), and from southern Siberia to northern China (10.9%), and Eurasia (21.7%). The most diverse beetle families were darkling beetles (Tenebrionidae, 18 species) and weevils (Curculionidae, 15 species) in planted and natural saxaul plots. There were three families (Cerambycidae, Histeridae, Trogidae) in the natural plots that were not collected in the planted plots.

**Figure 2.**
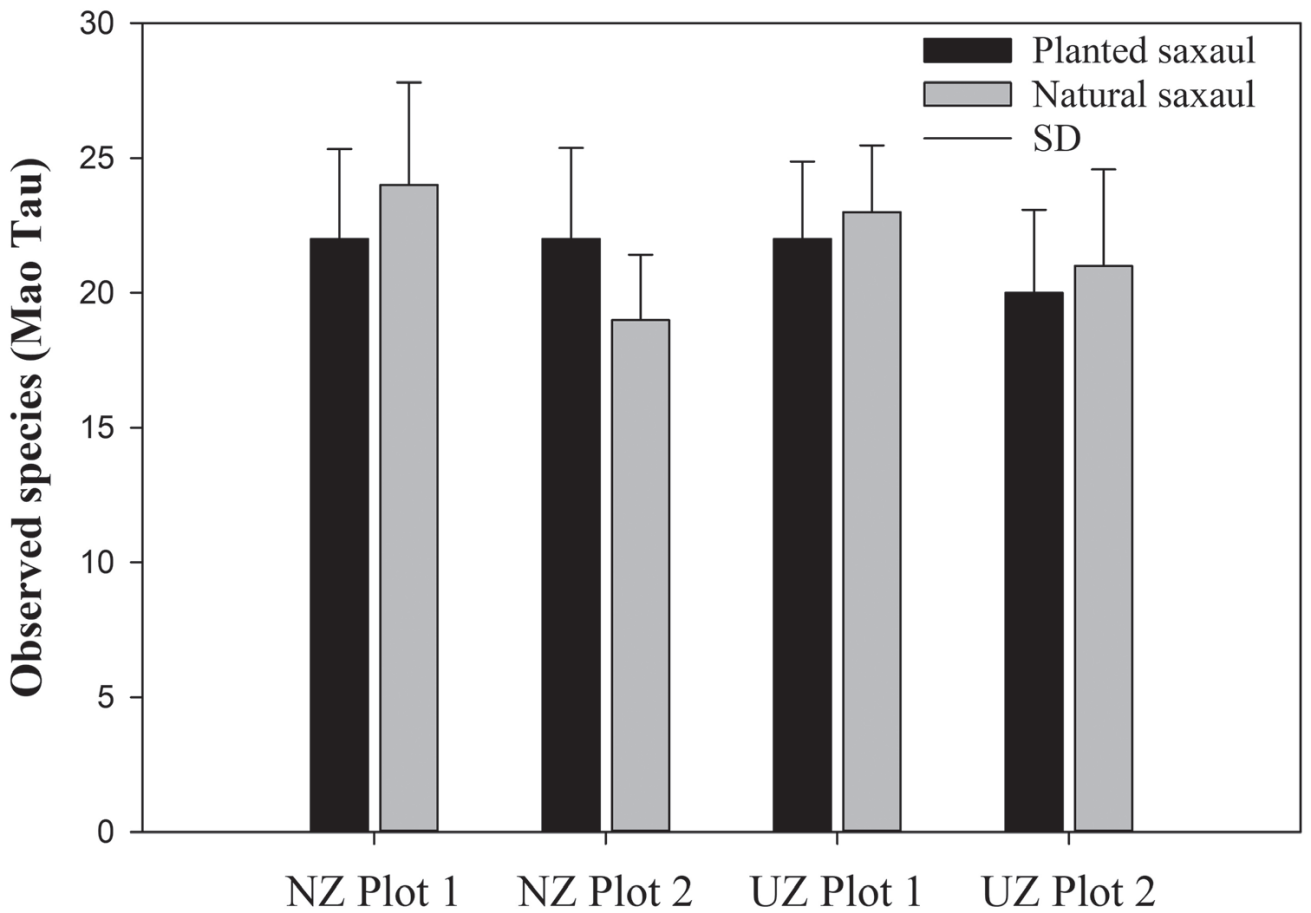
Mean observed species richness (Mao Tau) in planted and natural saxaul sites.

The observed species richness at natural saxaul plots ranged from 19 to 24 beetle species, whereas at the planted saxaul plots it ranged from 20 to 22 species (Fig. 2). The species richness at planted and natural saxauls were significantly different for the Ukhaa Zag site (T-value = −2.41, df = 10, *p* = 0.037 for plot 1 and T-value = −2.11, df = 10, *p* = 0.061 for plot 2). Herein, a weevil, *Temnorhinus oryx*, and a darkling beetle, *Anatolica sternalis gobiensis*, occurred only in natural plots of the Ukhaa Zag site.

**Figure 3.**
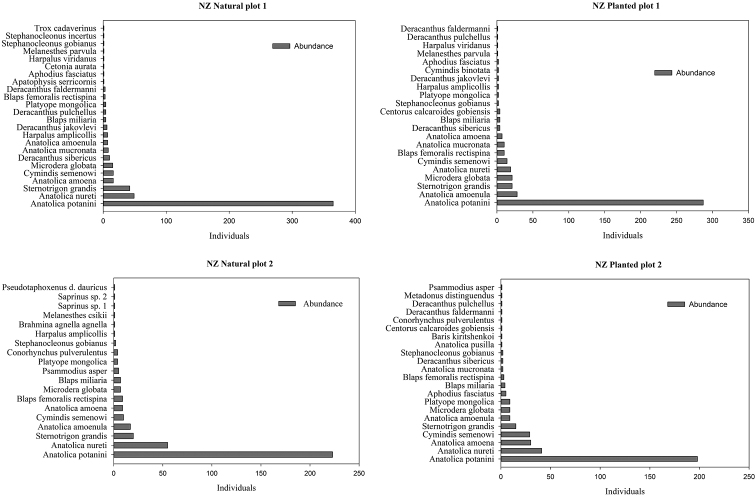
Abundances of species in the study plots of the Nariin Zag forest.

**Figure 4.**
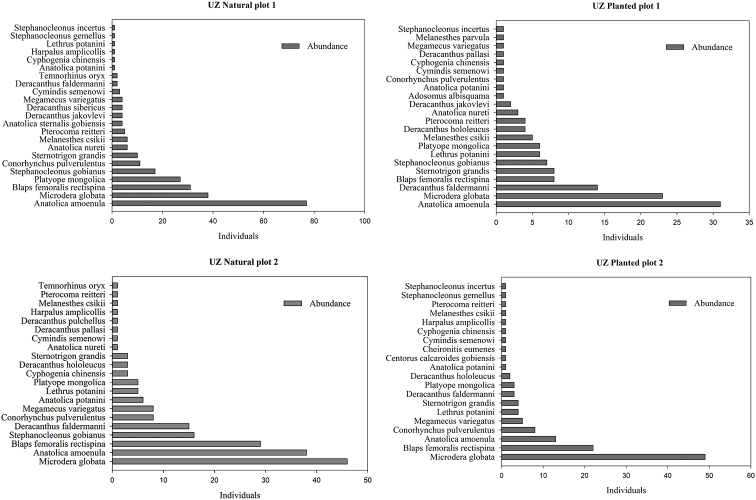
Abundance of species in the study plots of the Ukhaa Zag forest.

Seven to eight species in the natural plots and 10 to 11 species in the planted saxaul plots were represented by only one or two individuals (Table 1) (Figs 3, 4). The beetle species with the highest abundance in both natural and planted saxaul plots of the Nariin Zag forest was the darkling beetle *Anatolica potanini* (198–365 individuals) (Fig. 3). *Microdera globata* (23–47 individuals) and *Anatolica amoenula* (49–77 individuals) was the most abundant species in the Ukhaa Zag forest (Fig. 4). There were significant differences of beetle abundances in natural and planted plots 1 (T-Value = −2.86, df = 10, *p* = 0.017) and 2 (T-Value = −2.73, df = 10, *p* = 0.021) of the Ukhaa Zag site.

The dominance of *Anatolica potanini* at the plots in the Nariin Zag forest influenced the diversity index values and evenness of the plots. Therefore, species diversity and evenness at the plots were lower than at the Ukhaa Zag site. We observed significant differences in species diversity at planted and natural saxaul plots (Table 1). In general, species diversity indices of beetle communities at planted saxaul plots were higher than at natural plots.

**Figure 5.**
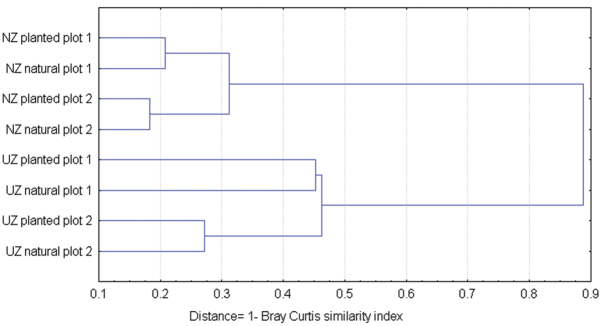
A dendrogram of beetle community dissimilarity.

Species composition of the beetle communities showed a dissimilarity (Fig. 5) between plots. The highest differences in beetle communities were between planted plot 1 and the corresponding natural plot in the Ukhaa Zag forest (45% dissimilar).

**Table 1.** Diversity parameters of beetle communities in planted and natural saxaul sites.

Sites	Plots	Singletons Mean	Doubletons Mean	Shannon index	Simpson index	Evenness	T-test, p value*
**NZ Plot 1**	Planted	4	6	1.55	2.34	0.50	6.94
	Natural	8	0	1.52	2.33	0.47	*p* = 0.000
**NZ Plot 2**	Planted	8	3	1.75	3.12	0.56	−17.71
	Natural	6	1	1.58	2.66	0.53	*p* = 0.000
**UZ Plot 1**	Planted	9	1	2.50	8.900	0.80	−2.77
	Natural	6	2	2.34	6.93	0.74	*p* = 0.020
**UZ Plot 2**	Planted	10	1	2.08	4.87	0.69	3.52
	Natural	8	0	2.32	7.50	0.76	*p* = 0.008

*T-test was calculated on Shannon diversity index.

## Discussion

Natural saxaul (*Haloxylon ammodendron*, black saxaul) forests in the Gobi Desert provide and sustain several beetle species associated with the tree and its environment. In our study, we found several of these species, including a long-horn beetle, *Apatophysis serricornis* (Gebler, 1843), which is known to feed on generative organs and roots of saxaul in desert areas of south-eastern Kazakhstan (Mombayeva et al. 2016). We also found a darkling beetle, *Cephogenia chinensis* (Faldermann, 1835), which was previously recorded in desert habitats with saxaul and *Eumylada punctifera punctifera* (Reitter, 1889) and had been found in crevices of the “burial mound” of saxaul, which is a sand compaction around a saxaul with high concentration of salt (Medvedev 1990).

A particular surprise was the complete absence of leaf beetles (Chrysomelidae) in our samples. Medvedev and Roginskaya (1988) listed 20 species of leaf beetles feeding on *Haloxylon* species. However, only six of them were reported from Mongolia: *Clytra atraphaxidis punctata* Weise, 1890 (Cryptocephalinae, Clytrini; Regalin and Medvedev 2010), *Pachybrachis fimbriolatus* (Suffrian, 1848), *Stylosomus major* Breit, 1919 (both Cryptocephalinae, Cryptocephalini; Schöller et al. 2010), *Ischyronota conicicollis* (Weise, 1890), *I. desertorum* (Gebler, 1833), *I. schusteri* Spaeth, 1914 (Cassidinae; Borowiec and Sekerka 2010). Adults of the three *Ischyronota* species might not have fallen off the trees when these were shaken and beaten, as adult cassidines cling firmly to the substrate when they are disturbed. However, their larvae would have fallen down, and we would certainly not have overlooked them, due to their characteristic appearance (Świętojańska and Borowiec 2007). Likewise, the three cryptocephalines would have dropped from the shaken trees, as it is their usual escape behaviour. The most plausible explanation of the complete lack of these species in our samples is that they are entirely absent in the Gobi Region of Mongolia. These species have been predominantly recorded from western and south-western Mongolia (Medvedev and Voronova 1976, 1977, 1979; Lopatin 1966, 1967, 1968, 1970).

The guild of herbivorous Coleoptera is well represented by the numerous weevil species (Appendix 1). The beetle community of the Nariin Zag forest is characterized by the presence of many rare species that were represented by one or two individuals and the dominance of a single darkling beetle species, *Anatolica potanini* Reitter, 1889, which is the main representative of sandy habitats with saxaul in the Mongolian desert (Medvedev 1990; Pfeiffer and Bayannasan 2012).

The total beetle abundance of the Ukhaa Zag plots is clearly lower than that of the Nariin Zag site. Although there is no absolute dominant species in the Ukhaa Zag plots, two darkling beetle species, *Microdera globata* (Faldermann, 1835) and *Anatolica amoenula* Reitter, 1889, were relatively highly abundant in both planted and natural plots. The genus *Microdera* is known as a geochortobiont living in surface soil and specializes in climbing up plants to eat seeds (Ulikpan 1993). *Anatolica amoenula* Reitter, 1889 is a typical species in places in the Gobi where brown soil dominates (Tsendsuren 1963). In the Ukhaa Zag forest, species of the genus *Deracanthus* were diverse, with a higher abundance than at the Nariin Zag site. They prefer flat sand surfaces rather than loose sands (Ulikpan 1993).

There was no significant difference between beetle species richness and abundance of planted and natural saxaul plots in the Nariin Zag habitat. This could be explained by the proximity of the planted plots, which were situated within the natural forest (Fig. 1), allowing beetles to re-occupy the area quickly. The age of the planted plots probably played a role. The saxaul plantation in the Nariin Zag forest was planted in 2013, and its growth success was 60–88.2% by 2016. According to an evaluation in 2018, vegetation diversity and richness of planted plots did not differ from the natural forest (Oyu Tolgoi LLC 2018). On the other hand, plantation of the Ukhaa Zag site was done in 2016, and its beetle species richness in the natural plots were significantly higher than the planted plots. Succession of invertebrates from natural to planted habitats was influenced by plantation age (Jansen 1997; Taki et al. 2010).

The higher values of diversity indices and species richness in the planted plots are driven by the presence of rare species, represented by only one or two individuals. However, a positive effect of reforestation on species richness of ground beetles was also observed, indicating that the creation of the pioneer habitats is important for successful reforestation (Januschke and Verdonschot 2016).

Ulikpan (1993) found a high level of endemism of beetles in the Gobi Desert. We likewise found that 17% of the recorded species were endemic to Mongolia.

In summary, restored saxaul forests (*Haloxylon ammodendron*) in the Gobi Desert are capable of supporting a natural beetle community composed mainly of species of Tenebrionidae and Curculionidae and characterised by a high level of endemism. Therefore, rehabilitation of saxaul, a native and a key plant species, is an important effort to conserve Gobi’s natural state and its living components. Our study is the first effort to evaluate the success of the saxaul rehabilitation programme in Mongolia using a beetle community.

## Appendix 1

**Table:** List of beetle species of natural and planted saxaul forests in the South Gobi Desert, Mongolia.

Species	Nariin Zag		Ukhaa Zag	
	Natural	Planted	Natural	Planted
** Carabidae **	Specimens (captured by pitfall traps)			
*Cymindis binotata* Fischer von Waldheim, 1820		2		
*Cymindis semenowi* Jakovlev, 1889	26	43	4	2
*Harpalus amplicollis* Ménétriés, 1848	8	2	2	1
*Harpalus viridanus* Motschulsky, 1844	1	1		
*Pseudotaphoxenus dauricus dauricus* (Fischer von Waldheim, 1823)	1			
** Cerambycidae **				
*Apatophysis serricornis* (Gebler, 1843)	1			
** Curculionidae **				
*Adosomus albisquama* Ter-Minasian, 1976				1
*Baris kiritshenkoi* Zaslavskij, 1956		1		
*Conorhynchus pulverulentus* (Zoubkoff, 1829)	4	1	19	9
*Deracanthus faldermanni* (Faldermann, 1835)	3	2	17	17
*Deracanthus hololeucus* (Faldermann, 1835)			3	6
*Deracanthus jakovlevi jakovlevi* Suvorov, 1908	6	2	4	2
*Deracanthus pallasi* Faust, 1890			1	1
*Deracanthus pulchellus* Gyllenhal, 1840	4	2	1	
*Deracanthus sibericus* (Thunberg, 1799)	10	6	4	
*Megamecus variegatus* (Gebler, 1830)			12	6
*Metadonus distinguendus* (Boheman, 1842)		1		
*Stephanocleonus gemellus* Voss, 1967			1	1
*Stephanocleonus gobianus* Suvorov, 1912	3	4	33	7
*Stephanocleonus incertus* Ter-Minasian, 1972	1		1	2
*Temnorhinus oryx* (Reitter, I897)			3	
** Geotrupidae **				
*Lethrus potanini* Jakovlev, 1889			6	10
** Histeridae **				
*Saprinus* sp. 1	1			
*Saprinus* sp. 2	1			
** Scarabaeidae **				
*Aphodius fasciatus* (Olivier, 1789)	1	7		
*Cetonia aurata* (Linnaeus, 1761)	1			
*Cheironitis eumenes* (Gebler, 1860)				1
*Brahmina agnella agnella* (Faldermann, 1835)	1			
*Psammodius asper* (Fabricius, 1775)	5	1		
** Tenebrionidae **				
*Anatolica amoena* (Faldermann, 1835)	25	37		
*Anatolica amoenula* Reitter, 1889	24	37	115	44
*Anatolica mucronata* Reitter, 1889	8	12		
*Anatolica nureti* Schuster & Reymond, 1937	104	60	7	3
*Anatolica potanini* Reitter, 1889	588	485	7	2
*Anatolica pusilla* Kaszab, 1967		1		
*Anatolica sternalis gobiensis* Kaszab, 1964			4	
*Blaps femoralis rectispina* Kaszab, 1968	12	13	60	30
*Blaps miliaria* Fischer von Waldheim, 1844	11	8		
*Centorus calcaroides gobiensis* (Kaszab, 1964)		5		1
*Cyphogenia chinensis* (Faldermann, 1835)			4	2
*Eumylada punctifera punctifera* (Reitter, 1889)*				
*Melanesthes csikii* Kaszab, 1965	1		7	6
*Melanesthes parvula* Kaszab, 1967	1	1		1
*Microdera globata* (Faldermann, 1835)	22	30	84	72
*Platyope mongolica* Faldermann, 1835	8	11	32	9
*Pterocoma reitteri* Frivaldszky, 1889			6	5
*Sternotrigon grandis* (Faldermann, 1835) (= *S. mongolica* Reitter, 1889)		36	13	12
** Trogidae **				
*Trox cadaverinus* Illiger, 1802	1			

*Species captured only by tree searching.
